# A pilot study of cerebral metabolism and serotonin 5-HT_2A_ receptor occupancy in rats treated with the psychedelic tryptamine DMT in conjunction with the MAO inhibitor harmine

**DOI:** 10.3389/fphar.2023.1140656

**Published:** 2023-09-28

**Authors:** Klemens Egger, Frederik Gudmundsen, Naja Støckel Jessen, Christina Baun, Sandra N. Poetzsch, Vladimir Shalgunov, Matthias M. Herth, Boris B. Quednow, Chantal Martin-Soelch, Dario Dornbierer, Milan Scheidegger, Paul Cumming, Mikael Palner

**Affiliations:** ^1^ Department of Psychiatry, Psychotherapy and Psychosomatics, Psychiatric University Hospital Zurich, University of Zurich, Zurich, Switzerland; ^2^ Neuroscience Center Zurich, University of Zurich and Swiss Federal Institute of Technology Zurich, Zurich, Switzerland; ^3^ Department of Nuclear Medicine, Bern University Hospital, Bern, Switzerland; ^4^ Department of Clinical Research, University of Southern Denmark, Odense, Denmark; ^5^ Department of Nuclear Medicine, Odense University Hospital, Odense, Denmark; ^6^ Department of Drug Design and Pharmacology, University of Copenhagen, Copenhagen, Denmark; ^7^ Department of Forensic Pharmacology and Toxicology, Zurich Institute of Forensic Medicine, University of Zurich, Zurich, Switzerland; ^8^ Department of Clinical Physiology, Nuclear Medicine and PET, Copenhagen University Hospital, Copenhagen, Denmark; ^9^ Department of Psychology, University Fribourg, Fribourg, Switzerland; ^10^ School of Psychology and Counselling, Queensland University of Technology, Brisbane, Australia; ^11^ Neurobiology Research Unit, Copenhagen University Hospital, Copenhagen, Denmark

**Keywords:** psychedelics, ayahuasca, pharmahuasca, DMT, harmine, serotonin receptor, [18 F]FDG-PET, PKPD

## Abstract

**Rationale:** The psychedelic effects of the traditional Amazonian botanical decoction known as ayahuasca are often attributed to agonism at brain serotonin 5-HT_2A_ receptors by *N,N*-dimethyltryptamine (DMT). To reduce first pass metabolism of oral DMT, ayahuasca preparations additionally contain reversible monoamine oxidase A (MAO-A) inhibitors, namely β-carboline alkaloids such as harmine. However, there is lacking biochemical evidence to substantiate this pharmacokinetic potentiation of DMT in brain via systemic MAO-A inhibition.

**Objectives:** We measured the pharmacokinetic profile of harmine and/or DMT in rat brain, and tested for pharmacodynamic effects on brain glucose metabolism and DMT occupancy at brain serotonin 5-HT_2A_ receptors.

**Methods:** We first measured brain concentrations of harmine and DMT after treatment with harmine and/or DMT at low sub-cutaneous doses (1 mg/kg each) or harmine plus DMT at moderate doses (3 mg/kg each). In the same groups of rats, we also measured *ex vivo* the effects of these treatments on the availability of serotonin 5-HT_2A_ receptors in frontal cortex. Finally, we explored effects of DMT and/or harmine (1 mg/kg each) on brain glucose metabolism with [^18^F]FDG-PET.

**Results:** Results confirmed that co-administration of harmine inhibited the formation of the DMT metabolite indole-3-acetic acid (3-IAA) in brain, while correspondingly increasing the cerebral availability of DMT. However, we were unable to detect any significant occupancy by DMT at 5-HT_2A_ receptors measured *ex vivo*, despite brain DMT concentrations as high as 11.3 µM. We did not observe significant effects of low dose DMT and/or harmine on cerebral [^18^F]FDG-PET uptake.

**Conclusion:** These preliminary results call for further experiments to establish the dose-dependent effects of harmine/DMT on serotonin receptor occupancy and cerebral metabolism.

## 1 Introduction

Ayahuasca refers to a plant-based psychedelic decoction used by certain indigenous peoples of South America for ritual, spiritual, and healing purposes as part of traditional Amazonian Medicine (Riba et al., 2001; Berlowitz et al., 2018). According to a widely held understanding, the psychedelic effects of botanical ayahuasca result from the synergism of two main constituents, whereby inhibition of monoamine oxidase type-A (MAO-A) by the β-carboline alkaloids harmine and harmaline from one plant (e.g., *Banisteriopsis caapi*) augments the oral bioavailability of the classical psychedelic compound *N,N*-dimethyltryptamine (DMT) from another plant (e.g., *Psychotria viridis*). The second most abundant β-carboline compound in *Banisteriopsis caapi* is tetrahydroharmine (THH), which can comprise 3%–47% of the total alkaloid content (Rivier and Lindgren, 1972). THH is a weak MAO-A inhibitor (Callaway et al., 1999) and is also a blocker of the plasma membrane serotonin transporter (SERT) (Buckholtz and Boggan, 1977), which may contribute to the overall psychopharmacology of botanical ayahuasca (Brito-da-Costa et al., 2020). The psychedelic effects of DMT have been proposed to be mainly mediated through agonism at brain serotonin 5-HT_2A_ receptors (McKenna et al., 1984), despite having only moderate *in vitro* affinity (K_i_ 127–1200 nM, IC_50_ 75–360 nM) (Lyon et al., 1988; Pierce and Peroutka, 1989; McKenna et al., 1990; Keiser et al., 2009; Ray, 2010). Receptor binding at serotonin 5-HT_1A_ (K_i_ 183 nM, IC_50_ 170 nM) and 5-HT_2C_ receptors (K_i_ 360–2630 nM, IC_50_ 360 nM), and other receptor types may contribute to the overall psychoactive effects of DMT (Lyon et al., 1988; Pierce and Peroutka, 1989; McKenna et al., 1990; Smith et al., 1998; Aghajanian and Marek, 1999; Nichols, 2004; Cozzi et al., 2009; Keiser et al., 2009; Ray, 2010). Treatment with ketanserin, a relatively specific 5-HT_2A_ receptor antagonist, substantially blunted the neurophysiological and subjective effects reported by ayahuasca participants, suggesting a major but not necessarily exclusive action via 5-HT_2A_ receptors (Valle et al., 2016). However, DMT has low efficacy by oral administration due to its rapid metabolism by MAO-A in the gut and other tissues (Barker, 2018). In some traditional ayahuasca preparations, co-administration of the β-carboline MAO-A inhibitor harmine seemingly potentiates the bioavailability and pharmacodynamic effects of DMT by blocking first pass metabolism in the gut. This model inspired the concept sometimes known as “pharmahuasca”, in which the effects of ayahuasca may be mimicked by co-administration of synthetic DMT and an MAO-A inhibitor such as harmine or moclobemide (Ott, 1999). However, there has been no formal demonstration of the potentiation of cerebral DMT concentrations by MAO-A inhibition with harmine, nor is there any investigation showing the cerebrometabolic effects of DMT/harmine or their associated target engagement at serotonin 5-HT_2A_ receptors in living brain.

Compared to DMT/harmine, there is a better understanding of the pharmacokinetic and pharmacodynamic principles of psychedelic action for psilocybin, or rather its active metabolite psilocin, which induces subjective effects comparable to those of ayahuasca (Nichols, 2016). In particular, a pioneering positron emission tomography (PET) study showed significant occupancy of psilocybin at serotonin 5-HT_2A_ receptors in the brain of human volunteers, using the antagonist radiotracer [^18^F]altanserin (Hasler et al., 2009b), which was recently confirmed in a PET study using the agonist ligand [^11^C]CIMBI-36 (McCulloch et al., 2022). In a PET study with the glucose analogue [^18^F]fluorodeoxyglucose ([^18^F]FDG) in healthy volunteers, acute challenge with psilocybin evoked widespread cerebral hypermetabolism ranging from 15% to 25% above baseline values (Vollenweider et al., 1997). Another study showed more nuanced cerebrometabolic effects of psilocybin, with 5%–10% increases in frontal operculum, anterior cingulate cortex, and inferior temporal cortex, along with 5%–10% decreases in the precentral cortex and thalamus (Gouzoulis-Mayfrank et al., 1999). There is almost no comparable information about the occupancy of DMT at brain serotonin receptors or the cerebrometabolic effects of harmine/DMT, either in synthetic formulations or as traditional botanical ayahuasca (Riba et al., 2006).

Two studies reported increased DMT concentrations and DMT half-life in rat brain and other tissues after administration of the irreversible MAO inhibitors iproniazid or tranylcypromine (Wang Lu and Domino, 1976; Sitaram et al., 1987). However, there are no comparable studies with a reversible MAO inhibitor such as harmine. Given this lack of basic knowledge, we undertook experiments in rats aiming to establish the pharmacological potentiation of DMT by conjoint administration of harmine. We measured the cerebral concentrations of harmine, DMT, and its metabolites indole-3-acetic acid (3-IAA), *N-*methyltryptamine (NMT), and DMT-*N-*oxide after subcutaneous administration of harmine and/or DMT at low (1 mg/kg each) or moderate doses (3 mg/kg each). We hypothesized that co-administration of harmine would potentiate the brain concentration of DMT as compared to the DMT only group. In the same groups of animals, we also assessed *ex vivo* the occupancy by DMT at serotonin 5-HT_2A_ receptors labelled with [^3^H]ketanserin or the more specific ligand [^18^F]MH.MZ. Here, we tested the hypothesis that harmine pretreatment would promote the displacement of exogenous 5-HT_2A_ receptor ligands by DMT via competitive binding *in vivo*. Furthermore, we undertook a pilot [^18^F]FDG-PET examination in the group of rats with low dose of harmine/DMT, aiming to establish the power of the method to detect perturbations in cerebral energy metabolism after the pharmacological challenge.

## 2 Materials and methods

### 2.1 Animals

Forty-three (43) male Long Evans rats (N = 24, 227 ± 12 g, for experiment 1; and N = 19, 268 ± 24 g, for experiment 2; purchased from Janvier Labs, Le Genest-Saint-Isle, France) were used in this study. The animals had *ad libitum* access to food and water on a 12-h light/dark cycle (lights were on from 6 a.m. to 6 p.m). Housing was in groups of four in individually ventilated cages with environmental enrichment, which consisted of wooden sticks, bedding and a red-colored shelter. We moved the animals to the PET facility at least 1 day before scanning to allow for acclimation. At the PET facility, the animals were housed in conventional cages supplied with dust-free bedding (alpha dri^®^, Shepherd Specialty Papers, Richland, Michigan, US) and kept at 22°C. Animals were fasted overnight prior to [^18^F]FDG-PET experiments. All animal procedures were conducted in accordance with the FELASA guidelines and with approval from The Danish Animal Experiments Inspectorate.

### 2.2 Chemicals

DMT and harmine, both as free base, were purchased from a commercial vendor (Cayman Chemicals, Ann Arbor, Michigan, US). [^3^H]Ketanserin (molar activity 844 MBq/µmol) was purchased from Perkin Elmer (Waltham, Massachusetts, US). All chemicals were stored at −20°C until use. [^18^F]MH.MZ was prepared at the Cyclotron Unit in the Department of Clinical Physiology, Nuclear Medicine and PET (Rigshospitalet) by alkylating MDL105725 with 2-[^18^F]fluoroethyl tosylate ([^18^F]FEtTos) in an automated synthesis module (Herth et al., 2008, 2009). Radiochemical purity of [^18^F]MH.MZ was >97% and molar radioactivity was 15–30 GBq/µmol at end of synthesis. For the first experiment, DMT was dissolved in ethanol (96%) at a concentration of 10 mg per mL and kept at −20°C until use; on the experimental day, the DMT stock solution was mixed with nine volumes of PBS, yielding a DMT concentration of 1 mg/mL solution in 10% EtOH/PBS. The slightly milky final DMT formulation was kept at 5°C in the dark until administration at a dose of 1 mg/kg body weight (s.c). Harmine was suspended in ethanol (96%) at a concentration of 1.5 mg per mL, which was then heated in a water bath at 90°C until dissolved. This solution was stored at −20°C until use, whereupon the stock solution was heated, resuspended and diluted 1:10 with PBS yielding a harmine concentration of 0.15 mg/mL 10% EtOH/PBS solution. After mixing, we noted some formation of needlelike crystals, which were heated and resuspended before injection. For the second experiment, DMT and harmine were both dissolved at 15 mg/mL in EtOH containing fumaric acid (1 mg/mL) and then diluted 1:10 with PBS to yield a final clean concentration of 1.5 mg/mL.

### 2.3 Experiment 1: Metabolic analysis, receptor occupancy and cerebral metabolism of harmine + DMT (1 mg/kg each)

**FIGURE 1:**
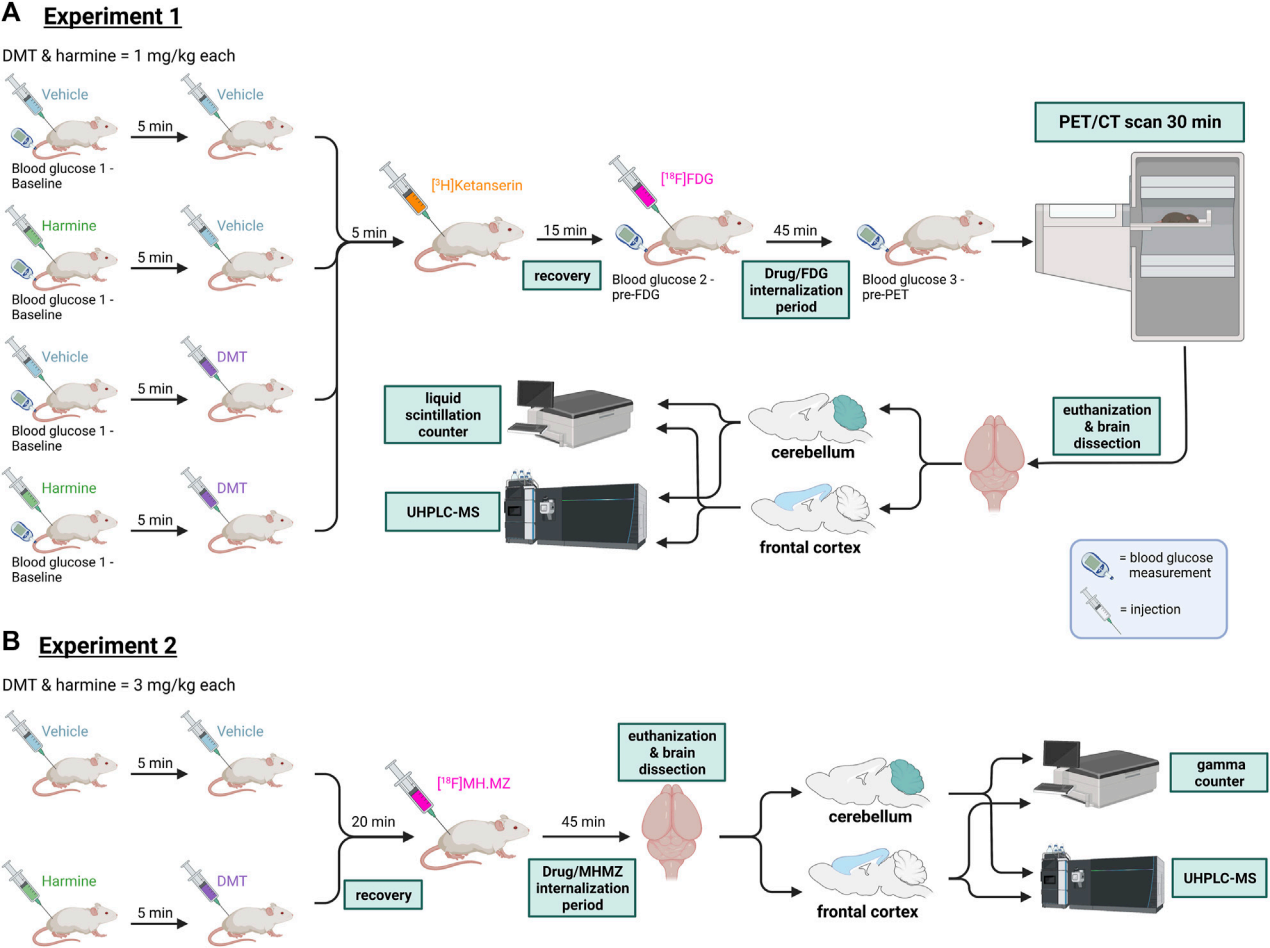
Experimental workflow in Experiments 1 and 2. **(A)** Experiment 1: Rats received either 2 × vehicle, harmine and vehicle, vehicle and DMT, or harmine and DMT (s.c.), then received successive injections of [^3^H]ketanserin (i.v.) and [^18^F]FDG (i.p). They were then allowed to roam freely for 45 min whereupon they were rapidly anesthetized for a PET/CT recording lasting 30 min. Next, rats were euthanized and brain samples (cerebellum and frontal cortex) were taken to measure [^3^H]ketanserin content by liquid scintillation counting, and the cerebral concentrations of harmine, DMT, and the metabolite 3-IAA by HPLC-MS. **(B)** Experiment 2: Rats received either 2 × vehicle, or harmine and DMT (s.c.), followed by [^18^F]MH.MZ injection (i.p). Rats were then allowed to roam freely for 45 min and then euthanized. Samples from their brain (cerebellum and frontal cortex) were taken to measure serotonin receptor occupancy/competition with a gamma counter and DMT, harmine and their metabolite concentrations with UHPLC-MS. For details, please refer to the text. DMT = *N,N*-dimethyltryptamine, [^18^F]FDG = [^18^F]fluorodeoxyglucose, PET = positron emission tomography. CT = computed tomography, UHPLC-MS = ultra-high performance liquid chromatography-mass spectroscopy. Figure created with BioRender.com.

Groups of (n = 6) rats received either saline, harmine (1 mg/kg), DMT (1 mg/kg), or harmine + DMT (1 mg/kg each) as subcutaneous injections, as indicated in Figure 1. Approximately 5 minutes later, the animals were lightly anesthetized using (2% isoflurane in pure oxygen) and received [^3^H]ketanserin (300 kBq; 740 MBq/µmol) as a bolus injection in 0.2 mL saline via a tail vein. Five minutes after this treatment, we administered [^18^F]FDG (9.8 ± 1.1 MBq) in 0.2 mL saline (i.p), and then returned the rats to a holding cage. At 45 min after [^18^F]FDG administration, rats were anesthetized (2% isoflurane in pure oxygen) and placed feet first in a prone position on a heated animal bed (70 mm) inside the aperture of the tomograph for a PET/CT recording on a Siemens INVEON PET/SPECT/CT multimodality pre-clinical scanner in docked mode (Siemens, Knoxville, TN, US). Blood glucose levels for the correction of [^18^F]FDG PET uptake were measured via tail vein puncture at three time points using a handheld glucometer (Bayer, Contour Next, blood glucose monitoring kit) in a micro blood sample (5–10 µL): at baseline before harmine or vehicle injection, prior to [^18^F]FDG administration, and immediately before starting the PET recording. Respiration and temperature of the animal were monitored during the imaging session using the BioVet system (M2M Imaging, Cleveland, Ohio, US). First, for attenuation correction and anatomic orientation, a CT scan was performed with full rotation in 270-degree projections, 4×4 bins, and low magnification, with reconstruction using the Feldkamp algorithm, a Sheep-Logan filter, slight noise reduction, and Hounsfield calibration. Next, a 30 min dynamic PET recording was obtained starting approximately 50 min post-injection of [^18^F]FDG, with energy window set at 350–650 keV and axial scan length of 127 mm. At the conclusion of the PET recording, rats were sacrificed while still anesthetized, thus 95 min after drug administration. Terminal samples of cerebellum (non-binding reference tissue) and frontal cortex were transferred to preweighed 7 mL liquid scintillation vials, reweighed, and added with 0.9 mL of aqueous based solubilizer (Solvable, Perkin Elmer) for dissolution during 48 h at room temperature. Next, 7 mL of liquid scintillation counting cocktail (Ultima Gold™ XR, Perkin Elmer) was added to each sample, and the content of tritium (i.e., [^3^H]ketanserin, cpm/mg) was measured by liquid scintillation counting (TRI-CARB, Perkin Elmer). We calculated the occupancy by DMT at frontal cortex 5-HT_2A_ receptors for the three treatment groups relative to the specific binding ratio ([cortex-cerebellum]/cerebellum) for the control group.

### 2.4 Experiment 2: Metabolic analysis and receptor occupancy of harmine + DMT (3 mg/kg each)

As shown in Figure 1, N = 19 rats received an injection of saline (n = 14) or harmine (n = 5) at a dose of 3 mg/kg (s.c.), followed 5 minutes later by DMT at a dose of 3 mg/kg. Animals were lightly sedated using 2% isoflurane in pure oxygen at 20 min post DMT injection, whereupon they received a 0.2 mL intravenous injection of [^18^F]MH.MZ diluted in saline to a radiochemical concentration of approximately 20 MBq/mL. Rats then returned to their home cage with withdrawal of anesthesia. After 45 min, rats were sacrificed by decapitation, and the brains dissected into cerebellum and frontal cortex samples for radioactivity measurement and chemical analysis, thus 65 min after harmine + DMT. The gamma-activity in weighed samples of cerebellum and frontal cortex regions was counted with a Packard Cobra gamma counter (GMI, Ramsey, Minnesota, US), as previously reported (Bærentzen et al., 2019). As with [^3^H]ketanserin, the occupancy at [^18^F]MH.MZ binding sites in frontal cortex was calculated from the specific binding ratio (cortex-cerebellum)/cerebellum) measured in the saline control and treatment groups.

### 2.5 [^18^F]FDG PET image reconstruction and analysis

The dynamic PET recordings were histogrammed into six frames of 5 minutes each, and reconstructed with attenuation, decay, and scatter correction using the OSEM3D/SP-MAP algorithm (2 x OSEM iterations and 18 x MAP iterations) with a matrix size of 128 × 128 pixels, resulting in a final target resolution of 0.8 mm/pixel. Pre-processing of PET scans was performed using PMOD v4.2 (PMOD Technologies LLC, Zurich, Switzerland). The [^18^F]FDG images were averaged across the six frames and then converted into standardized uptake values (SUV), whereupon the image was cropped to the head of the animal and automatically co-registered to a standard [^18^F]FDG-PET template. We used Schiffer’s atlas (Schiffer et al., 2006) for parcellation of bilateral brain regions within the cortico-striato-thalamo-cortical (CSTC) circuit, i.e. bilateral nucleus accumbens (NAc), medial prefrontal cortex (mPFC), orbitofrontal cortex (OFC), hippocampus, striatum, thalamus, visual cortex, cerebellum as a comparator region, and whole brain. We selected these regions from the CSTC circuit based on a conceptual model holding that the thalamus acts as a filter/gatekeeper of sensory information, broadcasting to cortical regions that subserve conscious experience (Wang et al., 2006; McAlonan et al., 2008). Perturbation of this CSTC circuit is implicated in the action of psychedelic compounds (Geyer and Vollenweider, 2008; Preller et al., 2019). We scaled individual regional SUV results to the mean blood glucose levels measured during the recording session (SUVgluc) and to the global [^18^F]FDG uptake (SUVglob).

### 2.6 Chemical analysis of brain tissue

DMT standard was purchased from Lipomed (Arlesheim, Switzerland), 3-IAA from Merck (Darmstadt, Germany) and harmine, DMT-d6, and harmine-d3 from TRC Chemicals (Toronto, Canada). All other used chemicals were of highest grade available. Brain samples from frontal cortex and cerebellum (each ca. 100 mg) were weighed and transferred into 2 mL tubes containing metal beads (MP Biomedicals, Illkirch, France), rapidly frozen on dry ice, and stored at −80°C for subsequent analysis of harmine, harmol, DMT, 3-IAA, NMT (*N*-methyltryptamine, and DMT-*N*-oxide. To all samples, 25 µL of internal standard (IS) mixture containing 40 ng/mL DMT-d6 and 40 ng/mL harmine-d3 and 25 µL of methanol were added. The brain samples were homogenized for 30 s at 30 Hz using a bench-top mill (Mixer Mill MM 400, Retsch, Haan, Germany). Samples were then shaken for 10 minutes and centrifuged for 5 minutes at 10,000 rpm. A 350 µL portion of the supernatant was transferred to an autosampler vial, evaporated to dryness under a gentle stream of nitrogen, and reconstituted in 50 µL H_2_O/ACN 95:5 v/v. Samples were analyzed on an ultra-high performance liquid chromatography (UHPLC) system (Shimadzu, Kyoto, Japan) coupled to a 5500 QTrap linear ion trap quadrupole mass spectrometer (Sciex, Darmstadt, Germany). The mobile phases consisted of a mixture of water (eluent A) and ACN (eluent B), both containing 0.1% formic acid (v/v). Injection volume was 2 µL. Using a Kinetex C18 column (50 × 2.1 mm, 2.6 µm) (Phenomenex, Aschaffenburg, Germany), the flow rate was set to 0.5 mL/min with the following gradient: start conditions 98% of eluent A, decreasing to 70% within 4 min followed by a decrease to 5% within 1 min. These conditions were held for 0.5 min, and then switched to the starting conditions for re-equilibration during 1.5 min. The mass spectrometer was operated in positive electrospray ionization mode with scheduled multiple reaction monitoring. The following quantifier and qualifier transitions were selected: m/z 189 → 58 and 189 → 115 for DMT, m/z 195 → 64 for DMT-d3, m/z 213 → 169 and 213 → 198 for harmine, m/z 216 → 170 for harmine-d3, m/z 176 → 130 and 176 → 77 for 3-IAA, m/z 205 → 144 and 205 → 117 for DMT-*N*-oxide, m/z 175 → 144 and 175 → 117 for NMT, and m/z 199 → 131 and 199 → 171 for harmol. For the reference and a representative chromatogram from one rat brain, see Supplementary Figure S4. External calibrator and quality control (QC) samples were prepared using water spiked with 25 µL calibration spiking solution and 25 Ll IS-mixture. The samples were extracted following the same procedure mentioned above. Calibration range was 0.5–500 ng/g for DMT and DMT-N-oxide, 2.5–120 ng/g for harmine, 35–3500 ng/g for 3-IAA, 1–80 ng/g for harmol, and 0.015–10 ng/g for NMT. The regressions were calculated using a linear or quadratic model with 1/X weighting.

### 2.7 Statistics

Brain concentration/occupancy data and [^18^F]FDG-PET results were analyzed with the StatsModel (v. 0.13.5) and Pingouin (v. 0.5.2) packages in Python (Seabold and Perktold, 2010). For experiment 1, we used one-way ANOVA, or the Kruskal-Wallis-test for [^18^F]FDG-PET uptake in brain regions violating the assumption of data normality. No corrections for multiple testing were performed in this exploratory experiment, which is intended to inform a follow-up [^18^F]FDG-PET study. In thalamus (SUVglob), the Dunn’s *post hoc* test with Sidak correction for multiple comparisons served to test which of the four groups differed in [^18^F]FDG uptake. Group-wise mean blood glucose concentrations for normalization of PET data were tested for differences between time-points with repeated-measures ANOVAs. Specific binding ratios from the occupancy data were compared group-wise with one-way ANOVA for experiment 1 ([^3^H]ketanserin occupancy) and with a Mann-Whitney U test for experiment 2 ([^18^F]MH.MZ occupancy), given the non-normally distributed control sample. We used paired *t*-tests to test differences in the brain DMT concentrations between frontal cortex and cerebellum in the rats that received harmine and DMT in both experiments; these results were uncorrected for multiple comparisons.

Normal distribution of occupancy data, blood glucose levels, [^18^F]FDG-PET data in each brain region were checked with the Shapiro-Wilk-test and visually confirmed by Q-Q-plots. Levene’s test served to test violations of the assumption of homogeneity of variance in the data. Given the 2 × 2 design of Experiment 1 and the small sample size per group (n = 6), this [^18^F]FDG-PET study should be considered exploratory. The significance level of a single group comparison was set to *p* < 0.05, with no corrections for multiple testing.

### 2.8 Data availability

Raw and processed data and additional scripts to recreate tables and plots is available at the project’s OSF repository (https://osf.io/dn8fy/).

## 3 Results

### 3.1 Harmine inhibits metabolism and increases cerebral availability of DMT

In Table 1 we report brain concentrations of harmine, DMT, and 3-IAA in frontal cortex and cerebellum from experiment 1 (1 mg/kg DMT and/or harmine) and experiment 2 (3 mg/kg DMT + harmine). Given the very low detected concentrations of the DMT metabolites NMT and DMT-N-oxide and the harmine metabolite harmol, we present these results in Supplementary Figure S2 and S3. Molar concentrations of DMT and its metabolites are shown in Supplementary Table S1 for all groups of rats that received DMT from both experiments.

**TABLE 1:** Brain concentrations of harmine, DMT, and 3-IAA in frontal cortex and cerebellum.

		Experiment 1 (1 mg/kg)				Experiment 2 (3 mg/kg)	
Compound	Region	Veh	Har	DMT	Har + DMT	Veh	Har + DMT
DMT	Cerebellum	5.9 (6.3)	4.1 (5.1)	8.7 (3.6)	105.1 (89.2)	0.0 (0.0)	1472.7 (369.2)
	Front Cort	10.8 (8.7)	4.8 (4.4)	14.5 (13.6)	149.5 (123.2)	0.0 (0.0)	2122.7 (434.2)
3-IAA	Cerebellum	13.3 (3.7)	11.1 (1.9)	398.5 (128.3)	194.1 (117.2)	20.9 (2.1)	530.7 (129.8)
	Front Cort	20.4 (9.5)	14.1 (1.2)	503.9 (137.7)	257.5 (103.6)	49.1 (11.4)	652.1 (186.5)
Harmine	Cerebellum	1.7 (3.0)	103.9 (64.8)	13.6 (31.0)	189.0 (134.3)	0.0 (0.0)	411.6 (137.8)
	Front Cort	0.8 (0.6)	128.4 (65.0)	20.1 (46.0)	325.6 (345.6)	0.0 (0.0)	435.9 (107.4)

Values in columns 3–8 are presented as mean (SD) in ng/g. In experiment 1, n = 5 for group ‘Veh’ and n = 6 for groups ‘Har’, ‘DMT’, and ‘Har + DMT’. In experiment 2, n = 4 for group ‘Veh’ and n = 5 for group ‘Har + DMT’. Veh = vehicle, Har = harmine, DMT = *N,N*-dimethyltryptamine, 3-IAA, indole-3-acetic acid, Front Cort = frontal cortex.

In experiment 1, the mean harmine concentration in brain tissue was 116.2 ng/g (0.55 µM) after harmine alone and 257.3 ng/g (1.21 µM) after harmine + DMT. After DMT alone, the concentration of 3-IAA was 503.9 ng/g (2.9 µM) in frontal cortex and 398.5 ng/g (2.3 µM) in cerebellum, *versus* 257.5 ng/g (1.5 µM) in frontal cortex and 194.1 ng/g (1.1 µM) in cerebellum after harmine + DMT (1 mg/kg each). Only traces of DMT were detectable after DMT alone, *versus* 149.5 ng/g (0.8 µM) in frontal cortex and 105.1 ng/g (0.6 µM) in cerebellum after harmine + DMT (1 mg/kg each). A paired samples *t*-test in the latter group indicated a higher DMT concentration in the frontal cortex compared to cerebellum (*t* (5) = 2.75, *p*_uncorrected_= 0.04, 95% CI [2.91, 85.9], Cohen’s *d =* 0.41). DMT and harmine were only detectable in trace amounts in the control brain samples.

In experiment 2, the mean brain concentration of harmine was 423.8 ng/g (2.0 µM) in the harmine + DMT group (3 mg/kg each), thus 1.6-fold higher than after the lower harmine + DMT dose. The mean brain 3-IAA concentration was 652.1 ng/g (3.7 µM) in frontal cortex and 530.7 ng/g (3.0 µM) in cerebellum after harmine + DMT, thus 1.3-times higher than after the low harmine + DMT dose. The concentration of DMT was 2122.7 ng/g (11.3 µM) in frontal cortex and 1472.7 ng/g (7.8 µM) in cerebellum, thus 14-fold higher than after the lower harmine + DMT dose. A paired samples *t*-test revealed a significantly higher DMT concentration in the frontal cortex than in cerebellum; *t* (4) = 10.3, *p*_uncorrected_ *=* 5.03 × 10^−4^, 95% CI [476.7, 825.3], Cohen’s *d =* 1.62. Traces of 3-IAA (10–40 ng/g) were present in brain samples from the saline groups from experiments 1 and 2. Endogenous DMT and harmine were not detectable in control brain samples.

### 3.2 Occupancy at 5-HT_2A_ binding sites *ex vivo*

We made two independent attempts to establish DMT occupancy at cortical 5-HT_2A_ receptors following administration of harmine and DMT. In experiment 1, the cerebral uptake of [^3^H]ketanserin gave an overall mean specific binding ratio in frontal cortex of 1.9 ± 0.8 across groups (vehicle = 1.88 ± 0.73; harmine = 1.28 ± 1.09; DMT = 2.15 ± 0.39; harmine + DMT = 2.28 ± 0.62). One-way ANOVA revealed no statistically significant differences in frontal cortex specific binding ratios between the four groups, as measured at 95 min after DMT/harmine administration (1 mg/kg each; *F(3, 20)* = 2.09, *p* = 0.13, η^2^ = 0.24). In experiment 2, [^18^F]MH.MZ had an overall mean specific binding ratio of 3.8 ± 1.5 in frontal cortex in the saline group *versus* 4.4 ± 0.8 in the harmine + DMT group, as measured at 65 min after administration of harmine/DMT (3 mg/kg each). A two-sided Mann-Whitney U test did not indicate a significant group difference in frontal cortex specific binding ratio (U = 28.0, *p =* 0.56, common language effect size (CLES) = 0.4). Individual datapoints for both experiments are shown in Supplementary Figure S5.

### 3.3 [^18^F]FDG-PET

[^18^F]FDG-PET data from one rat in the control group was lost due to technical failure. Two different preprocessing approaches were used to inform sample size calculations for a later follow-up study.

#### 3.3.1 Approach 1: whole-brain normalization (SUVglob)

**FIGURE 2:**
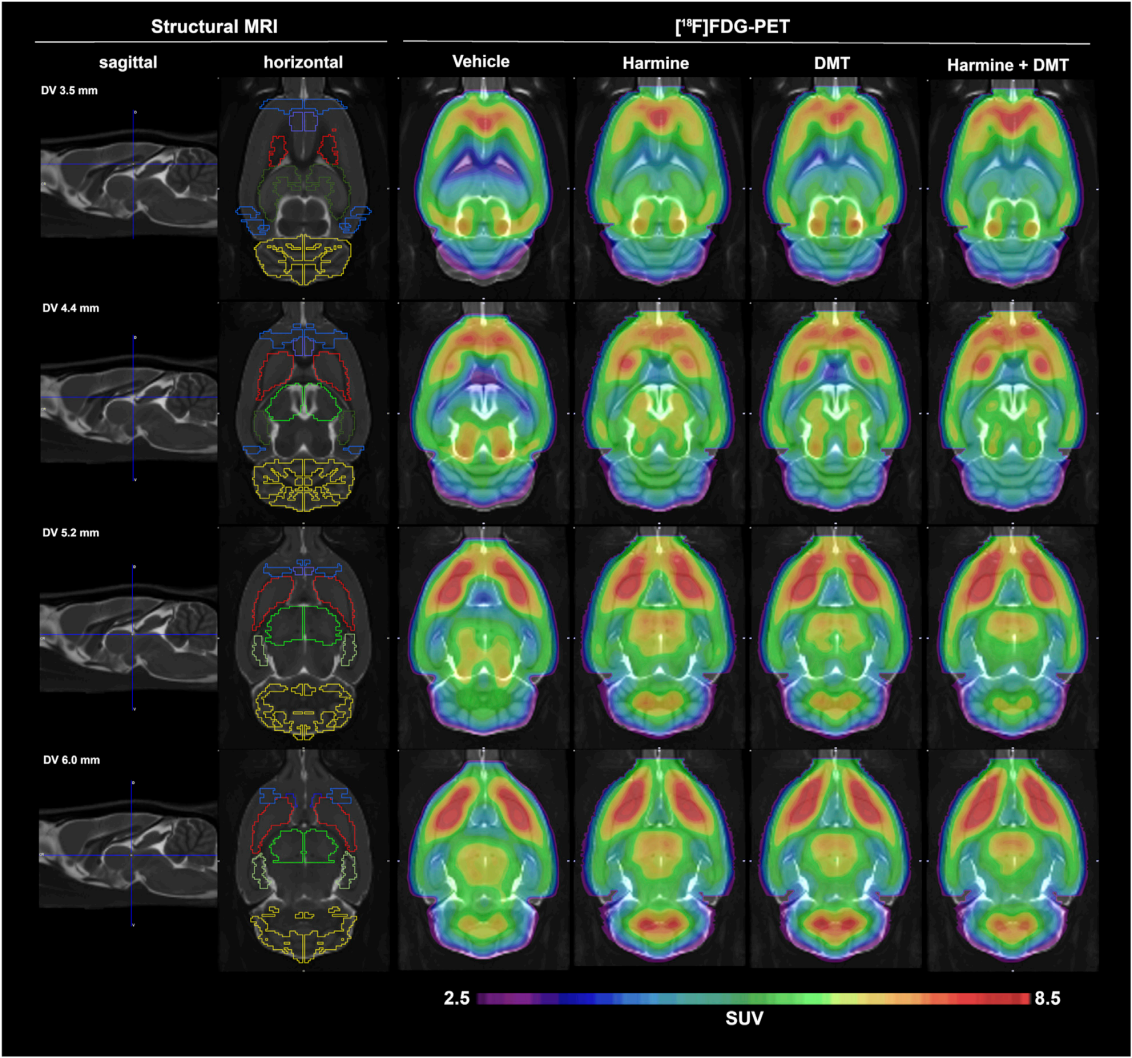
Mean globally normalized SUV (SUVglob) results from experiment 1. [^18^F]FDG-PET data in groups of (n = 5) control and (n = 6) rats treated with harmine, DMT, or harmine + DMT (1 mg/kg s.c. each). The first two columns display a structural MRI template that was used for parcelation of brain PET data. The second column depicts the regions of interest, namely (from rostral to caudal): orbitofrontal cortex (OFC, blue), medial prefrontal cortex (mPFC, purple), nucleus accumbens (NAc, dark blue), striatum (red), anterodorsal hippocampus (dark green), posterior hippocampus (light green), thalamus (neon green), visual cortex (blue), cerebellum (yellow). The SUVglob images represent the mean of six frames of 5 minutes duration, starting at 60 min after [^18^F]FDG administration.

Mean [^18^F]FDG SUV maps after whole brain normalization (SUVglob) are presented in Figure 2. Qualitatively, there were no conspicuous effects on brain [^18^F]FDG uptake of harmine and DMT, separately or in combination when administered at 1 mg/kg. Regional results for mean SUVglob values are presented in Table 2. In the whole-brain normalized data, the Kruskal–Wallis test was significant in the thalamus (χ^2^ (3) = 8.14, *p* = 0.04, η^2^ = 0.27). *Post-hoc* pairwise comparisons using Dunn’s test with Sidak *p*-value adjustment for multiple comparisons indicated that the harmine group was significantly different from the vehicle group (vehicle - harmine: Z = −2.66, *p* = 0.047). No other *post hoc* pairwise comparisons in thalamus showed a significant difference (*p*-value ranges from 0.18 to 0.99). Additionally, there was a trend for relative increases in [^18^F]FDG uptake in the hippocampus (Kruskal–Wallis test, χ^2^ (3) = 6.07, *p* = 0.11, η^2^ = 0.16). No other brain regions showed statistically significant differences in the whole-brain normalized data (see Table 2). SUVglob data are plotted in Supplementary Figure S6.

**TABLE 2:** [^18^F]FDG-PET standardized uptake values (SUV) normalized to whole-brain uptake (SUVglob) and group statistics.

Region	Veh	Har	DMT	Har + DMT	F/*Χ* ^2^	*p*	df	*η* ^2^
mPFC	7.11 (0.66)	7.09 (0.32)	6.95 (0.34)	7.16 (0.32)	0.29	0.83	(3, 19)	0.04
OFC	6.25 (0.45)	6.5 (0.44)	6.52 (0.15)	6.71 (0.4)	1.36	0.29	(3, 19)	0.18
visual cortex	6.3 (0.48)	6.1 (0.24)	6.27 (0.13)	6.18 (0.27)	0.52	0.67	(3, 19)	0.08
hippocampus	4.76 (0.91)	5.32 (0.23)	4.95 (0.21)	5.0 (0.21)	6.07	0.11	3	0.16
NAc	6.57 (0.53)	6.23 (0.18)	6.2 (0.36)	6.17 (0.28)	1.46	0.26	(3, 19)	0.19
striatum	6.92 (0.42)	6.99 (0.12)	6.91 (0.24)	7.17 (0.27)	1.13	0.36	(3, 19)	0.15
thalamus	6.08 (0.51)	6.54 (0.13)	6.33 (0.1)	6.37 (0.21)	8.14^a^	0.04	3	0.27
cerebellum	4.54 (0.58)	4.52 (0.15)	4.56 (0.23)	4.37 (0.29)	0.39	0.76	(3, 19)	0.06

Values in columns 2–5 represent mean (SD) in SUV, values per group, n = 5 for ‘Veh’ and n = 6 for ‘Har’, ‘DMT’, and ‘Har + DMT’. For hippocampus and thalamus Kruskal-Wallis-test (χ^2^) was used, for all other regions a one-way ANOVA (F) was used. The last three columns represent corresponding *p*-values, degrees of freedom, and effect size η^2^.

^a^ *p* < 0.05. mPFC, medial prefrontal cortex; OFC, orbitofrontal cortex; NAc = nucleus accumbens, Veh = vehicle, Har = harmine, DMT = N,N-dimethyltryptamine, df = degrees of freedom.

#### 3.3.2 Approach 2: blood glucose normalization (SUVgluc)

Mean plasma glucose concentrations in the four treatment groups at three time points during the PET examination were in the range of 5.7–7.0 mM (Supplementary Table S2). Repeated measures ANOVA did not indicate any main effects of vehicle, harmine, and/or DMT injections over time on plasma glucose levels (*p*-value ranges from 0.1 to 0.85; for details, see Supplementary Table S2). Therefore, we obtained SUVgluc by normalizing SUV levels to the individual mean plasma glucose measurement. Regional results for mean SUVgluc values are shown in Supplementary Table S3. There were no significant group differences of glucose metabolism in any brain region for SUVgluc data to one-way ANOVAs (for results, see Supplementary Table S3). SUVgluc data are plotted in Supplementary Figure S7.

## 4 Discussion

### 4.1 Harmine inhibits metabolism and increases cerebral availability of DMT

The present study confirmed the predicted effect of harmine to increase the brain concentrations of DMT. A complete dose-response study with increasing harmine dose with a fixed DMT dose and *vice versa* would be necessary to understand the dose-response relationship of DMT and harmine in full detail. However, only traces of DMT were present in brain, except when we administered DMT along with harmine (1 mg/kg and 3 mg/kg for both drugs). Previous pharmacokinetic studies of harmine in rats at doses ranging from 0.5 to 3.3 mg/kg i.v. and 20–40 mg/kg p.o., indicated a plasma half-life of 3.5 h, which would predict for persistent MAO-A inhibition in the present study (Guan et al., 2001; Li et al., 2017; Wang et al., 2019; Brito-da-Costa et al., 2020). We have elsewhere estimated the apparent affinity (K_D_) of [^18^F]fluoroethyl-harmol to be 300 nM at MAO-binding sites in living rat brain, as compared to 2 nM *in vitro* (Maschauer et al., 2015). Dopamine receptor ligands show similarly high discrepancies between ligand affinity *in vitro* and *in vivo* (Cumming, 2011), likely due to low free fractions in brain tissue, or other intrinsic factors related to compartmentation *in vivo*. Thus, given the 150-fold *in vitro*/*in vivo* affinity difference for [^18^F]fluoroethyl-harmol, the 2.0 nM K_D_ of [^11^C]harmine *in vitro* (Bergstrom et al., 1997), might predict an apparent affinity for harmine in the order of 300 nM for MAO-A in living brain. We now find brain harmine concentrations of approximately 1000 nM at 100 min after treatment with 1 mg/kg and 2000 nM at 70 min after the higher dose; assuming 300 nM affinity *in vivo*, it seems reasonable to expect a substantial inhibition of MAO-A in brain after harmine at present doses in the range of 1–3 mg/kg.

#### 4.1.1 DMT metabolism in rat brain

Previous reports emphasized the importance of MAO-A for peripheral metabolism of DMT, especially after oral administration. Present findings of high concentrations of 3-IAA in brain after administration of DMT alone are consistent with considerable penetration of DMT into brain, despite with very rapid and substantial deamination *in situ*, especially in visceral tissues and in brain neurons (McEwen Jr. et al., 1966; Barker et al., 1980). Our results highlight the role of brain MAO-A in moderating the pharmacokinetics of DMT (refer to Supplementary Figure S1 for known metabolic routes of DMT and harmine). The rapid oxidative deamination of DMT by MAO-A explains the short-acting effects when DMT is administered i.v. or via smoking (Erspamer, 1955). In a recent human study, the plasma half-life of DMT after i.v. administration with an infusion protocol over 90 min (doses ranging from 0.6 mg/min to 25 mg bolus + 1.0 mg/min) ranged from 5.0 to 5.8 min in the early phase (i.e., immediately after end of infusion) and from 14 to 16 min in a later stage starting around 16–18 min after stopping the infusion (Vogt et al., 2023). DMT attains a brain:blood partition ratio in rats of about 5:1 shortly after i.p. injection, but is rapidly cleared from brain and circulation (Cohen and Vogel, 1972; Sitaram et al., 1987). We assume that MAO-A in brain forms the acidic metabolite 3-IAA from DMT *in situ*, rather than deriving from the periphery. As such, the 50% lower cerebral 3-IAA concentrations after co-administration of harmine and DMT in experiment 1 may suggest 50% inhibition of MAO-A in living brain. Follow-up studies of the effects of probenecid treatment on harmine and DMT concentrations in blood and brain might confirm the relevance of deamination of DMT by brain MAO-A. We find that the extent of MAO-A inhibition sufficed to obtain brain DMT concentrations of approximately 0.8 µM after harmine + DMT at a dose of 1 mg/kg each, *versus* 11.3 µM after the higher doses. Data presented in Supplementary Table S1 indicate a DMT/3-IAA ratio in brain of approximately 0.53 in experiment 1 *versus* 2.83 in experiment 2; this suggests a substantially greater MAO-A inhibition with the three-fold higher harmine dose, which indeed resulted in two-fold higher brain concentrations of harmine. A greater degree of MAO-A inhibition likely accounts for the 14-fold higher DMT concentrations in brain, despite an only 3-fold higher DMT dose. We note that the slightly cloudy DMT solution in experiment 1 implies incomplete solvation, which might also have contributed to the non-linear dose-response of brain DMT concentration in experiment 2.

Previous work indicated that pretreatment with the irreversible MAO inhibitors iproniazid, tranylcypromine, and pargyline enhanced the bioavailability of DMT and increased the behavioral responses in rats (Wang Lu and Domino, 1976; Jenner et al., 1980), as likewise shown for 5-methoxy-DMT administered in conjunction with iproniazid (Sitaram et al., 1987). Similarly, harmaline and the MAO-A-preferring irreversible inhibitor clorgyline potentiated the behavioral effects of low doses of 5-methoxy-DMT in rats (Halberstadt et al., 2008). The present results may constitute the first formal demonstration of ayahuasca’s main pharmacological mechanism of action in brain, in showing a substantial potentiation of bioavailability of DMT in brain of harmine-treated animals. However, there is no consensus that DMT and harmine are the sole active constituents of botanical ayahuasca. As noted above, potential effects of SERT blockade by THH in plant decoctions should be a matter for future investigations. In present experiments 1 and 2, we unexpectedly found 50% higher DMT concentrations in frontal cortex compared to cerebellum; this cannot be due to 5-HT_2A_ binding, since the DMT concentrations in brain greatly exceeded the abundance of receptors in rat cortex via quantitative autoradiography (B_max_ circa 100 nM) (López-Giménez et al., 1997). Further experimentation may reveal the basis of this preferential uptake or retention in frontal cortex.

### 4.2 Serotonin 5-HT_2A_ receptor occupancy by DMT

DMT is a partial agonist at serotonin 1A, 2A, and 2C receptors (Nichols, 2016), as well as serotonin 1B, 1D, 2B, 5A, 6, and 7 receptor subtypes, with affinities *in vitro* ranging from 39 nM to 2.1 µM (Keiser et al., 2009). Agonism at serotonin 5-HT_2A_ receptors is thought to be a necessary but not sufficient factor accounting for the visual hallucinogenic effects of classical psychedelics such as LSD, psilocybin, and DMT (Carbonaro and Gatch, 2016; Carhart-Harris and Nutt, 2017; Canal, 2018). DMT has moderately high affinity for serotonin 5-HT_2A_ receptors *in vitro*, with K_i_ estimates ranging from 127 to 1200 nM and IC_50_ ranging from 75 to 360 nM (Lyon et al., 1988; Pierce and Peroutka, 1989; McKenna et al., 1990; Keiser et al., 2009; Ray, 2010). Despite the caveat raised above that affinities may differ considerably *in vitro* and *in vivo*, it seems reasonable to have expected some significant DMT occupancy at 5-HT_2A_ sites in brain of living rats. However, we were unable to detect any such occupancy in the present experiment 1 ([^3^H]ketanserin) or experiment 2 ([^18^F]MH.MZ), despite brain DMT concentrations as high as 11.3 µM, nearly 10-fold higher than the affinity of DMT for serotonin 5-HT_2A_ sites measured *in vitro*. We emphasize that, despite having conducted the occupancy measurement 95 min after the DMT or vehicle administration, we injected the [^3^H]ketanserin (experiment 1) or [^18^F]MH.MZ (experiment 2) only 5 or 20 min, respectively, after the drug administration. These time points correspond to the predicted peak DMT concentrations in blood when co-administered with a MAO inhibitor (Wang Lu and Domino, 1976; Jenner et al., 1980; Sitaram et al., 1987). This suggests that timing of 5-HT_2A_ radioligand administration was not likely a factor in the present negative results for occupancy *ex vivo*. Based on calculation from affinities, oral DMT (up to 0.85 mg/kg) and harmine (up to 3.4 mg/kg) administered to humans should give peak DMT occupancy (38%–45%) at brain 5-HT_2A_ receptors around 0.5–1.5 h post-administration (Koeck, 2015). There was very low uptake of [^3^H]ketanserin measured in rat brain at 95 min after intravenous injection, as reported previously in the rat for its fluorinated analogue [^18^F]altanserin (Riss et al., 2011). While this property of [^3^H]ketanserin would have disfavored the *ex vivo* detection of occupancy by DMT, we obtained concurring negative results with [^18^F]MH.MZ, a fluorinated analogue of MDL100907 (Herth et al., 2008), which is distinctly more sensitive than [^3^H]ketanserin/[^18^F]altanserin for detecting serotonin 5-HT_2A_ receptors in living rat brain (Hansen et al., 2013). Ketanserin blocks the subjective, sensomotoric, and cognitive effects of psychedelics such as LSD and psilocybin in humans (Quednow et al., 2012; Kometer et al., 2013; Preller et al., 2018). However, pretreatment with ketanserin (40 mg) gave only partial blockade of subjective and physiological effects of ayahuasca (Valle et al., 2016). Indeed, recent work with mescaline derivatives implicated the 5-HT_2C_ receptor in their hallucinogenic effects, which were very similar to those of classical psychedelic drugs (Custodio et al., 2022). A previous PET study with the partially selective serotonin 5-HT_2A_ antagonist ligand [^18^F]altanserin showed occupancy as high as 35% at 5-HT_2A_ receptors in human frontal cortex after challenge with psilocybin (Hasler et al., 2009a), whereas more recent work with the selective 5-HT_2A_ agonist ligand [^11^C]Cimbi-36 showed occupancy as high as 70% (Madsen et al., 2019). We did not systematically assess behavioral indices that might have confirmed psychoactive effects in rats of harmine/DMT at present doses. However, we did note that harmine-treated rats were calmer and needed less isoflurane to sustain anesthesia. We observed the characteristic dose-dependent head twitch response (Halberstadt et al., 2020; Odland et al., 2022) in some DMT- (and harmine-) treated rats. Thus, we have no ready explanation for the completely lacking evidence for significant occupancy by DMT at 5-HT_2A_ receptors. We speculate that DMT and the 5-HT_2A_ receptor antagonist ligands might not compete at identical binding domains, or that dynamic agonist-induced internalization processes might mask the occupancy. Testing these possibilities calls for further experiments, with consideration of the general difficulty in translating *in vitro* binding results to *in/ex vivo* scenarios. Intravenous DMT infusions administered over 90 min in humans built an acute tolerance with respect to psychedelic effects despite increasing plasma concentrations (Vogt et al., 2023), which again hints at pharmacodynamic behavior in the brain that is not exclusively explicable by the plasma concentrations. This scenario might fit with the above-mentioned potential mechanisms of receptor internalization or acute desensitization of serotonin receptors, which could attenuate DMT effects in the brain.

### 4.3 [^18^F]FDG-PET

Experiment 1 did not reveal any significant effects of harmine/DMT treatment on regional or global SUVgluc. There was a trend towards 5% relative increases in SUVglob in hippocampus and thalamus in the harmine and DMT groups, which only reached the target α-level of 0.05 when comparing the harmine with the control group in thalamus in the SUVglob data (Table 2). We administered [^18^F]FDG 20 min after DMT/harmine administration, aiming thereby to capture cerebral glucose metabolism during an interval that was maximally perturbed by the psychoactive compounds. Previous [^18^F]FDG-PET studies with psilocybin challenge in healthy humans indicated widespread increases in the cerebral metabolic rate for glucose (CMRglc) (Vollenweider et al., 1997), or more moderate and circumscribed increases and decreases in [^18^F]FDG uptake (Gouzoulis-Mayfrank et al., 1999). There are no previous studies of the cerebrometabolic effects of ayahuasca constituents, although two studies with, botanical ayahuasca showed increased cerebral perfusion rate in frontal and paralimbic areas in healthy subjects and in the nucleus accumbens, insula, and subgenual area in patients with depression (Riba et al., 2006; Sanches et al., 2016). Since DMT and psilocybin should share a common action via 5-HT_2A_ receptor agonism (Canal, 2018; Cumming et al., 2021), we expected cerebrometabolic activation in the CSTC circuit in the present rat [^18^F]FDG-PET study.

We suppose that the harmine/DMT dose in Experiment 1 may have been inadequate to perturb cerebral [^18^F]FDG uptake, also in consideration of the small sample size. Indeed, the 25% variances in the SUVgluc values presented in Supplementary Table S3 predict that a sample size of n = 16 per group would suffice to detect a 25% treatment effect on global [^18^F]FDG uptake, whereas the 10% variances in SUVglob predicts that n = 16 per group would suffice to detect 10% treatment effects in relative glucose metabolism. As such, present results thus inform the design of further PET studies intended to detect small regional effects of harmine/DMT on cerebral glucose metabolism in the rat.

### 4.4 Limitations

The main limitations of this study arise from the low sample size for the [^18^F]FDG-PET study and the rather low dose of 1 mg/kg harmine and/or DMT in experiment 1. In particular, the present group size of n = 6 did not support our prediction of increased cerebral glucose consumption upon harmine/DMT treatment, although the globally normalized results indicated trend level increases in thalamus and a trend-level increase in hippocampus. The administration of harmine and DMT as free bases were not optimal for s.c. injections, as the substances were not completely in solution in experiment 1. However, the rough dose-dependence of brain harmine concentrations between the two experiments is consistent with substantial uptake of harmine administered as free base. The post-treatment intervals may not have been optimal for detecting occupancy at 5-HT_2A_ sites *ex vivo*. We note that the sequential administration of up to four different compounds in experiment 1 may have disfavored the reliable determination of certain endpoints. Additionally, we plan to confirm the cerebral formation of the DMT metabolite 3-IAA in studies employing probenecid treatment, which blocks the efflux of 3-IAA and other acidic metabolites from brain.

### 4.5 Conclusion

In summary, present results indicate that harmine co-treatment substantially inhibits the deamination of DMT in brain of living rats, resulting in brain DMT concentrations of 0.8 μM at 95 min after a harmine/DMT dose of 1 mg/kg each, *versus* 11.7 μM at 60 min after harmine/DMT administration at 3 mg/kg each. The concomitant changes in the DMT metabolite 3-IAA in brain suggest that cerebral MAO-A activity is an important factor limiting the central action of DMT, since 3-IAA is an acidic metabolite generated *in situ*. Despite these high brain DMT concentrations, we failed in two separate experiments to detect any significant occupancy at cortical serotonin 5-HT_2A_ receptors *ex vivo*, which calls for further investigations aiming to establish the receptor target engagement of DMT in brain. The [^18^F]FDG-PET study in groups of six animals did not detect significant effects of harmine/DMT at 1 mg/kg on cerebral energy metabolism, but present results could inform the design of further PET studies intended to detect small regional effects of harmine/DMT on cerebral glucose metabolism in the rat.

## Data Availability

The datasets presented in this study can be found in online repositories. The names of the repository/repositories and accession number(s) can be found in the article/Supplementary Material.
