# Supplementary material for: A pilot study of cerebral metabolism and serotonin 5-HT2A receptor occupancy in rats treated with the psychedelic tryptamine DMT in conjunction with the MAO inhibitor harmine
Source: Front Pharmacol. 2023 Sep 28;14:1140656. doi: 10.3389/fphar.2023.1140656 (PMC10568461; doi:10.3389/fphar.2023.1140656)
Supplement: Supplementary file 1 [file DataSheet1.pdf]

Supplementary Material

Pharmacokinetic and Pharmacodynamic Interaction of the Ayahuasca Constituents Harmine and Dimethyltryptamine (DMT) in the Rat Brain

Klemens Egger[1,2,3*], Frederik Gudmundsen[4,5], Naja Støckel Jessen[4,5,6], Christina Baun[4,5], Sandra N. Poetzsch[7], Vladimir Shalgunov[6,8], Matthias M. Herth[6,8], Boris B. Quednow[1,2], Chantal Martin-Soelch[9], Dario Dornbierer[1], Milan Scheidegger[1,2], Paul Cumming[3,10†], and †Mikael Palner[4,5,11†]

*** Correspondence:** Klemens Egger: klemens.egger@bli.uzh.ch

†shared last authorship

1. Known DMT and harmine metabolic pathways

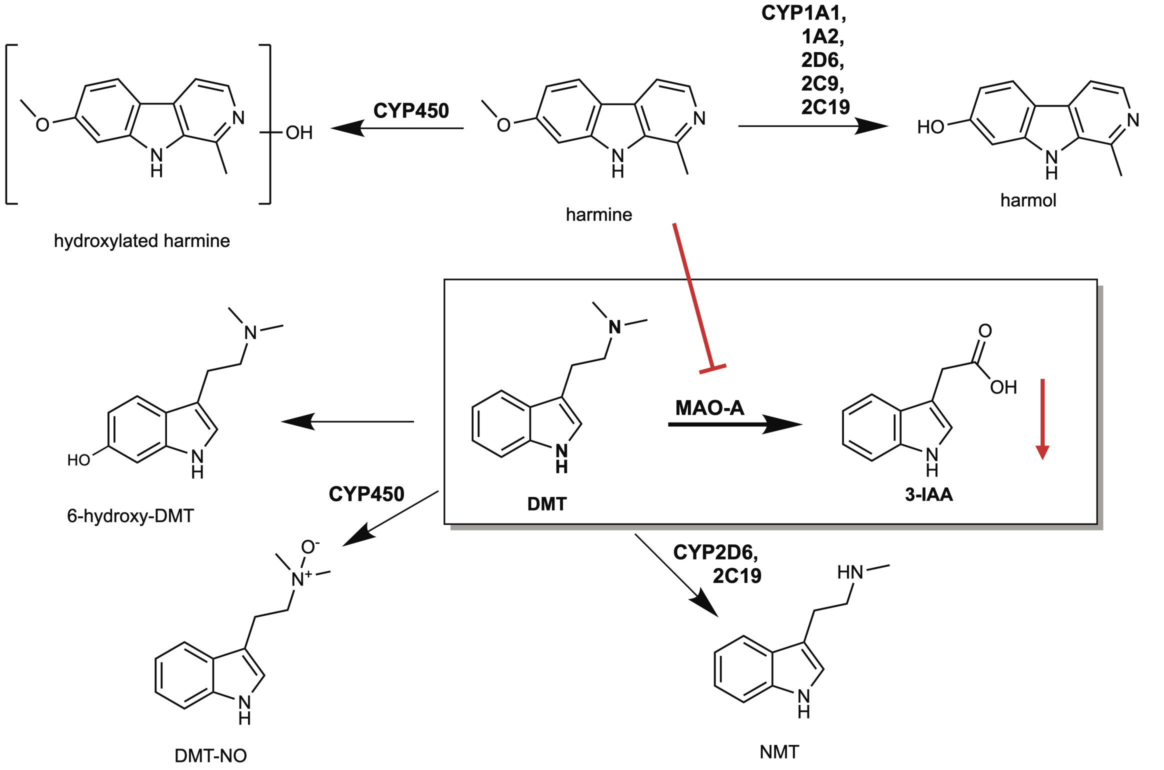

***Note: Reactions showing enzymatic metabolism by cytochrome P450 (CYP450), the exact enzyme isoforms involved in the metabolism are not yet established. The specific CYP450 isoforms are named wherever possible.**

Suppl. Fig. S1 Metabolites of *N,N*-dimethyltryptamine (DMT) and harmine, and effects of harmine on the metabolism of DMT. Upon administration, exogenous DMT rapidly undergoes oxidative deamination by monoamine oxidase A (MAO-A) in the gut and other tissues to yield indole-3-acetic acid (3-IAA), which is normally the main metabolic pathway (Barker, 2018). After MAO-A, the next most important enzyme for DMT metabolism is cytochrome P450 (CYP450), which degrades DMT to DMT-*N*-oxide (DMT-NO). Minor metabolic routes lead to the formation of *N*-methyltryptamine (NMT) or 6-hydroxy-DMT. Recent work shows that cytochrome oxidase isoforms CPY2D6 and CYP2C19 can also metabolise DMT to NMT (Caspar et al., 2018; Good et al., 2023). For the route to DMT-NO, the responsible isoforms are not yet established. When MAO-A is pharmacologically blocked (i.e., with harmine), the production of 3-IAA declines (red arrows) and the secondary metabolic routes become more important, such that formation of DMT-NO, NMT and 6-hydroxy-DMT increases (Brito-da-Costa et al., 2020). Harmine is metabolized in the body to hydroxy-harmine by CYP450 or to harmol by other enzymes in the cytochrome family (Brito-da-Costa et al., 2020).

2. Material for Brain Concentration Analysis

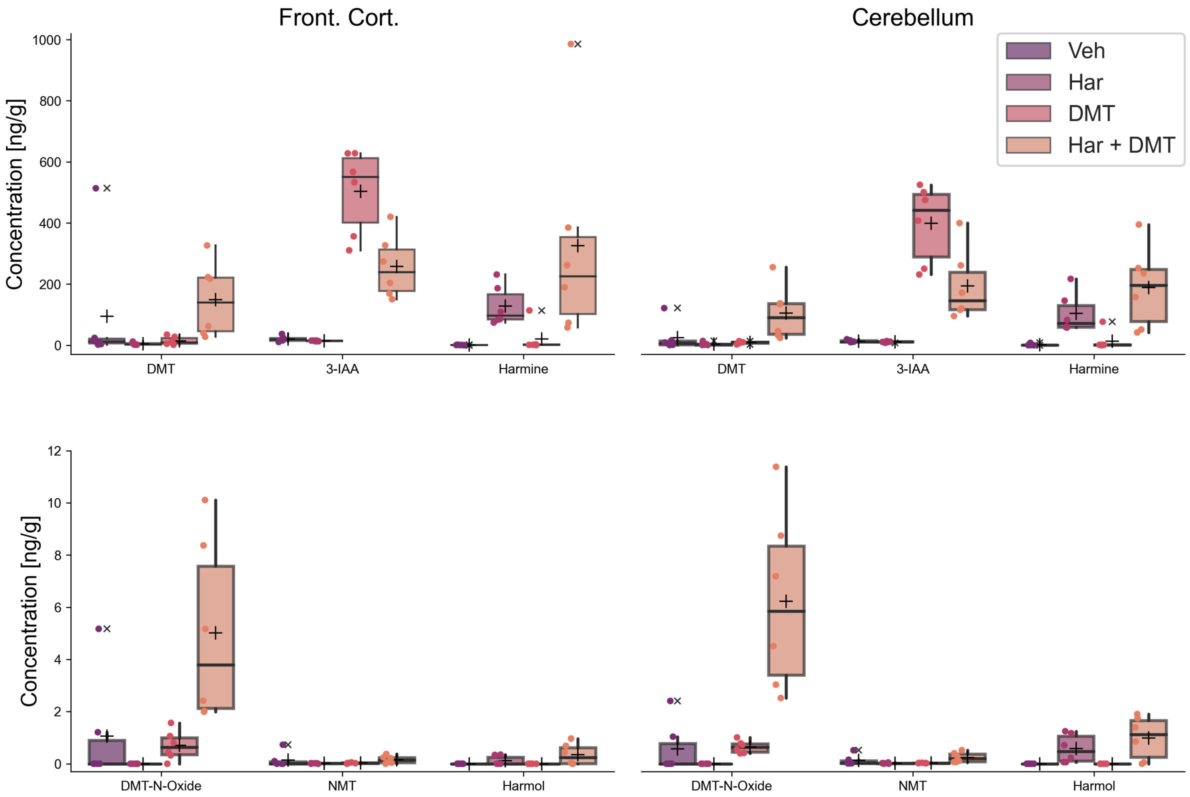

Suppl. Fig. S2 Brain concentrations of *N,N*-dimethyltryptamine (DMT) and harmine (Har) and their metabolites indole-3-acetic acid (3-IAA), *N*-methyltryptamine (NMT), DMT-*N*-oxide, and harmol in frontal cortex (column one) and cerebellum (column two) after dissection of the rats 95 min after drug administration in experiment 1. Administered doses were 1 mg/kg for Har and DMT. N=24; n=6 per group. Note: the outlier in the vehicle group was not included in the statistical calculations. + indicates mean, x indicates outliers.

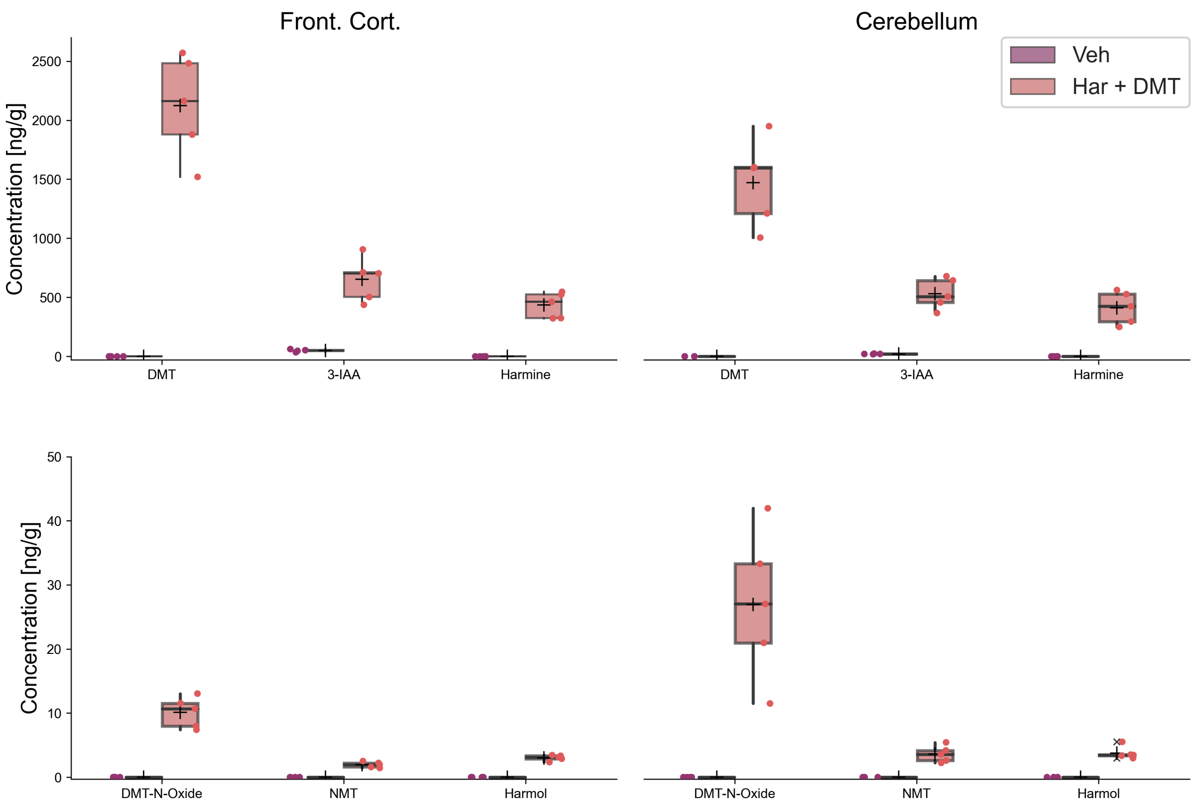

Suppl. Fig. S3 Brain concentrations of *N,N*-dimethyltryptamine (DMT) and harmine (Har) and their metabolites 3-indole-acetic-acid (3-IAA), *N*-methyltryptamine (NMT), DMT-*N*-oxide, and harmol in frontal cortex (column one) and cerebellum (column two) after dissection of the rats 65 min after drug administration in experiment 2. Administered doses were 3 mg/kg for Har and DMT. N=9; n=4 for vehicle and n=5 for Har + DMT. + indicates mean, x indicates outliers.

Suppl. Table S1 Molar concentrations (in nmol/L) and corresponding percentages of *N,N*-dimethyltryptamine (DMT) and its metabolites indole-3-acetic acid (3-IAA), *N*-methyltryptamine (NMT), DMT-*N*-Oxide.

Experiment	Group	Region	DMT	3-IAA	NMT	DMT-N-Oxide	Total mol. conc.
Exp 1	DMT	front cort	77.08 (2.6%)	2876.36 (97.3%)	0.16 (0.0%)	3.43 (0.1%)	2957
		cerbellum	45.98 (2.0%)	2274.61 (97.9%)	0.15 (0.0%)	3.16 (0.1%)	2324
		average	61.53 (2.3%)	2575.49 (97.5%)	0.15 (0.0%)	3.29 (0.1%)	2640
	Har + DMT	front cort	793.92 (34.7%)	1469.74 (64.2%)	0.89 (0.0%)	24.54 (1.1%)	2289
		cerbellum	557.98 (32.9%)	1107.88 (65.3%)	1.38 (0.1%)	30.49 (1.8%)	1698
		average	675.95 (33.9%)	1288.81 (64.7%)	1.14 (0.1%)	27.52 (1.4%)	1993
Exp 2	Har + DMT	front cort	11274.56 (74.9%)	3722.59 (24.7%)	11.15 (0.1%)	49.57 (0.3%)	15058
		cerbellum	7822.12 (71.1%)	3029.31 (27.5%)	20.66 (0.2%)	131.9 (1.2%)	11004
		average	9548.34 (73.3%)	3375.95 (25.9%)	15.9 (0.1%)	90.73 (0.7%)	13031

The total molar concentration was calculated as the sum of DMT and its metabolites per row. The table contains data from groups of rats that received DMT in both experiments.

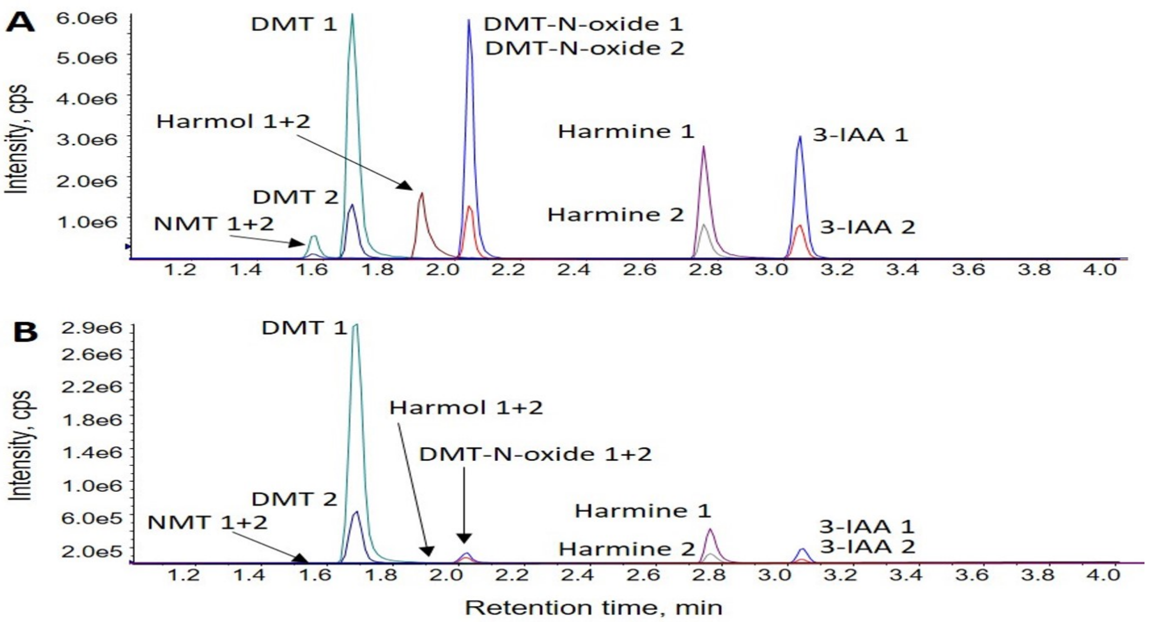

Suppl. Fig. S4 Extracted ion chromatograms of DMT 1 (189→58), DMT 2 (189→115), harmine 1 (213→169), harmine 2 (213→198), 3-IAA 1 (176→130), 3-IAA 2 (176→77), DMT-N-oxide 1 (205→144), DMT-N-oxide 2 (205→117), harmol 1 (199→131), harmol 2 (199→171), NMT 1 (175→144) and NMT 2 (175→117) in **A** an external calibration sample and **B** a rat brain sample.

3. Material for occupancy at 5-HT_{2A} binding sites ex vivo:

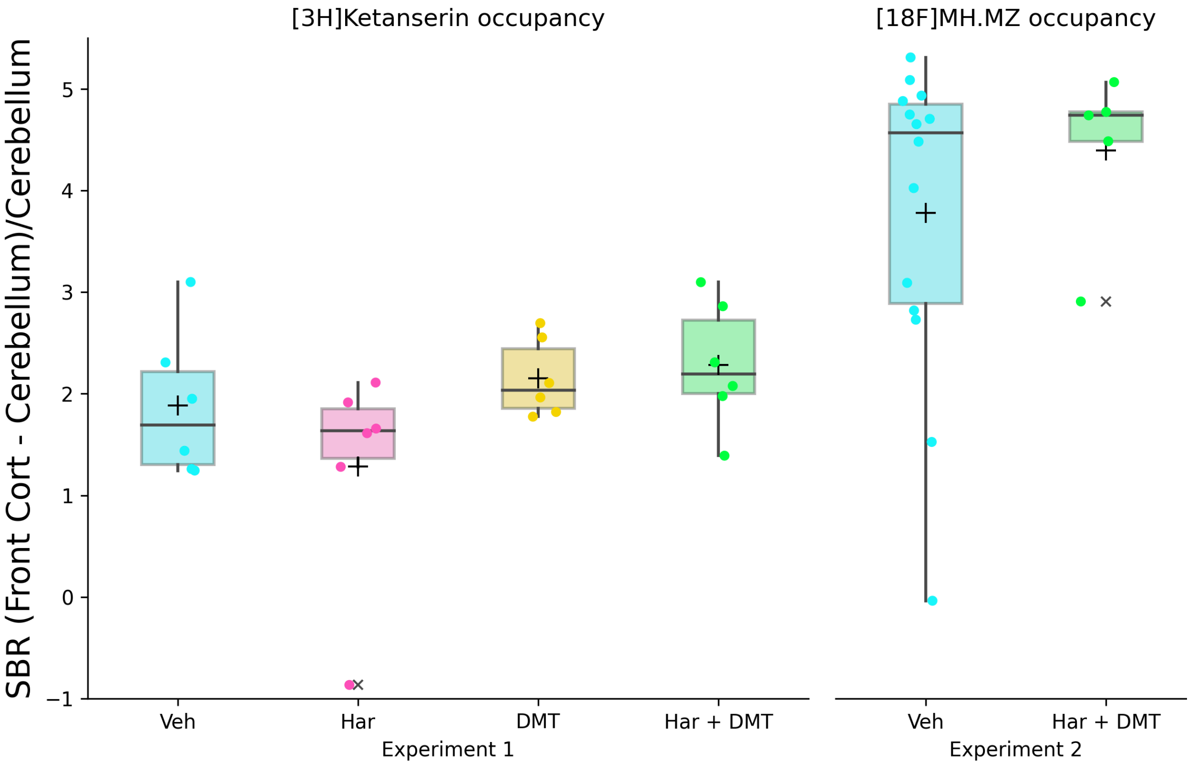

Suppl. Fig. S5 5-HT_{2A} receptor occupancy data from experiments 1 and 2. In experiment 1, DMT occupancy was measured with [^{3}H]ketanserin, in experiment 2 with the more specific radiotracer [^{18}F]MH.MZ. Frontal cortex specific binding ratio *ex vivo* was calculated from the radioactivity concentrations in frontal cortex and cerebellum as (frontal cortex - cerebellum)/cerebellum. There were no significant differences between the four groups in experiment 1 or the two groups in experiment 2. N=24; n=6 per group for experiment 1; and N=19; n=14 for veh group and n=5 for the Har + DMT group in experiment 2. + indicates mean, x indicates outliers.

SBR = specific binding ratio, Veh = vehicle, Har = harmine, DMT = *N,N*-dimethyltryptamine.

4. Material for [^{18}F]FDG-PET analysis

Suppl. Table S2 Plasma glucose concentrations in the four treatment groups in Experiment 1.

	**T1**	**T2**	**T3**	***F***	***p***	**df**	η^2
Veh	5.92 (0.74)	5.72 (0.48)	5.93 (0.93)	0.17	0.85	(2, 10)	0.02
Har	6.55 (0.73)	6.97 (0.7)	5.95 (0.91)	2.94	0.1	(2, 10)	0.25
DMT	6.17 (0.54)	5.87 (0.65)	6.33 (0.6)	0.97	0.41	(2, 10)	0.11
Har + DMT	6.37 (0.88)	5.78 (0.75)	6.38 (0.93)	2.09	0.17	(2, 10)	0.11

T1 = Timepoint 1, baseline at the time of the first injection (t = 0 min); T2 = Timepoint 2, glucose concentration at the time of [^{18}F]FDG injection (t + 25 min), T3 = Timepoint 3, glucose concentration before start of PET scan (t + 70 min), Veh = vehicle, Har = harmine, DMT = *N,N*-dimethyltryptamine, df = degrees of freedom.

Values in columns 2-4 represent mean (SD) glucose concentration in mmol/L per group, N=24; n = 6 for each group. The last four columns show the results from repeated-measures ANOVAs and their corresponding *p*-values, degrees of freedom, and effect size η^2.

Suppl. Table S3 [18F]FDG-PET standardized uptake values (SUV) normalized to whole-brain uptake (SUVglob) and group statistics.

Region	Veh	Har	DMT	Har + DMT	F	p	df	η^2
mPFC	7.26 (2.4)	7.28 (1.18)	7.3 (1.24)	6.67 (1.18)	0.24	0.87	(3, 19)	0.04
OFC	6.28 (1.59)	6.71 (1.33)	6.82 (0.84)	6.19 (0.61)	0.44	0.73	(3, 19)	0.06
visual cortex	6.4 (2.0)	6.29 (1.15)	6.57 (0.99)	5.75 (0.98)	0.44	0.73	(3, 19)	0.06
hippocampus	4.68 (1.12)	5.44 (0.77)	5.2 (0.85)	4.65 (0.69)	1.22	0.33	(3, 19)	0.16
NAc	6.63 (1.96)	6.4 (1.05)	6.48 (0.93)	5.75 (1.08)	0.53	0.66	(3, 19)	0.08
striatum	7.01 (2.15)	7.19 (1.21)	7.24 (1.07)	6.68 (1.17)	0.20	0.90	(3, 19)	0.03
thalamus	6.04 (1.38)	6.73 (1.15)	6.62 (0.86)	5.91 (0.83)	0.87	0.47	(3, 19)	0.12
cerebellum	4.44 (0.66)	4.62 (0.67)	4.76 (0.64)	4.03 (0.47)	1.62	0.22	(3, 19)	0.20
whole brain	5.56 (1.39)	5.69 (0.93)	5.8 (0.81)	5.14 (0.74)	0.52	0.67	(3, 19)	0.08

Values in columns 2-5 represent mean (SD) in SUV values per group. N=23; n = 5 for Veh and n = 6 for Har, DMT, and Har + DMT. For all regions a one-way ANOVA (F) was used. The last three columns represent corresponding p-values, degrees of freedom, and effect size η^2. Injected doses were 1 mg/kg harmine and/or DMT.
mPFC = medial prefrontal cortex, OFC = orbitofrontal cortex, NAc = nucleus accumbens, Veh = vehicle, Har = harmine, DMT = *N,N*-dimethyltryptamine, df = degrees of freedom.

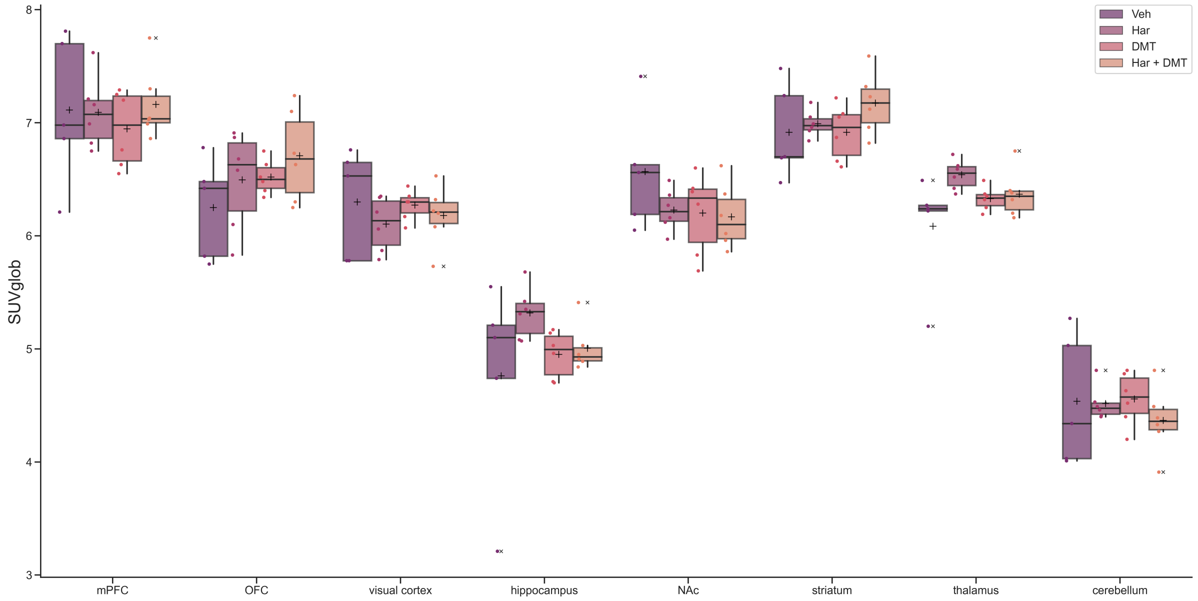

Suppl. Fig. S6 Boxplots and individual data points of [18F]FDG-PET whole-brain normalized standard uptake values (SUVglob) data from experiment 1.
N=23; n=5 for vehicle group, and n=6 for harmine, DMT and harmine + DMT group. Treatment groups are clustered per brain region. + indicates mean, x indicates outliers. Injected doses were 1 mg/kg harmine and/or DMT.
mPFC = medial prefrontal cortex, OFC = orbitofrontal cortex, NAc = nucleus accumbens, Veh = vehicle, Har = harmine, DMT = *N,N*-dimethyltryptamine.

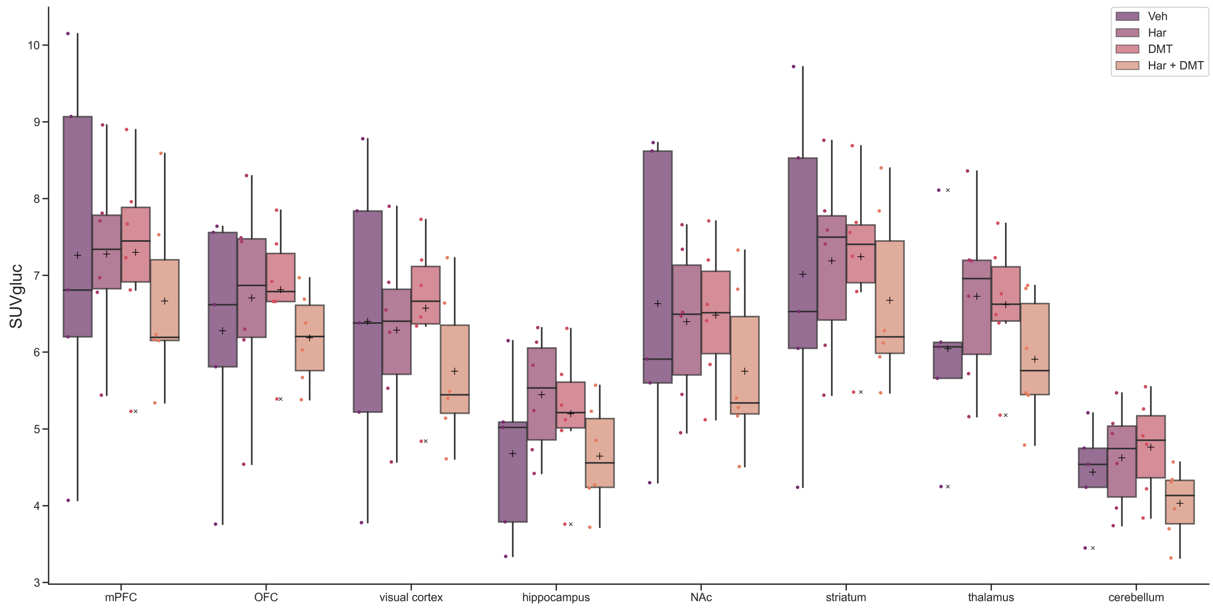

Suppl. Fig. 7 Boxplots and individual data points of [^{18}F]FDG-PET glucose normalized standard uptake values (SUVgluc) data from experiment 1.
N=23; n=5 for vehicle group, and n=6 for harmine, DMT and harmine + DMT group. Treatment groups are clustered per brain region. + indicates mean, x indicates outliers. Injected doses were 1 mg/kg harmine and/or DMT.
mPFC = medial prefrontal cortex, OFC = orbitofrontal cortex, NAc = nucleus accumbens, Veh = vehicle, Har = harmine, DMT = *N,N*-dimethyltryptamine.

References

Barker, S. A. (2018). N, N-Dimethyltryptamine (DMT), an Endogenous Hallucinogen: Past, Present, and Future Research to Determine Its Role and Function. *Front Neurosci* 12, 536. doi: 10.3389/fnins.2018.00536.

Brito-da-Costa, A. M., Dias-da-Silva, D., Gomes, N. G. M., Dinis-Oliveira, R. J., and Madureira-Carvalho, Á. (2020). Toxicokinetics and Toxicodynamics of Ayahuasca Alkaloids N,N-Dimethyltryptamine (DMT), Harmine, Harmaline and Tetrahydroharmine: Clinical and Forensic Impact. *Pharmaceuticals (Basel)* 13. doi: 10.3390/ph13110334.

Caspar, A. T., Gaab, J. B., Michely, J. A., Brandt, S. D., Meyer, M. R., and Maurer, H. H. (2018). Metabolism of the tryptamine-derived new psychoactive substances 5-MeO-2-Me-DALT, 5-MeO-2-Me-ALCHT, and 5-MeO-2-Me-DIPT and their detectability in urine studied by GC-MS, LC-MS, and LC-HR-MS/MS. *Drug Test Anal* 10, 184–195. doi: 10.1002/dta.2197.

Good, M., Joel, Z., Benway, T., Routledge, C., Timmermann, C., Erritzoe, D., et al. (2023). Pharmacokinetics of N,N-dimethyltryptamine in Humans. *Eur J Drug Metab Pharmacokinet* 48, 311–327. doi: 10.1007/s13318-023-00822-y.
